# Supplementary material for: High-Throughput Steady-State Enzyme Kinetics Measured in a Parallel Droplet Generation and Absorbance Detection Platform
Source: Anal Chem. 2022 Nov 23;94(48):16701–10. doi: 10.1021/acs.analchem.2c03164 (PMC9730296; doi:10.1021/acs.analchem.2c03164)
Supplement: Supplementary file 1 — ac2c03164_si_001.pdf [file ac2c03164_si_001.pdf]

Supporting Information

for

High-throughput steady-state enzyme kinetics measured in a parallel droplet generation and absorbance detection platform

Stefanie Neun[1], Liisa van Vliet[1], Florian Hollfelder[1,*], Fabrice Gielen[2,*]

[1]*Department of Biochemistry, University of Cambridge, 80 Tennis Court Road, Cambridge, CB2 1GA, UK*

[2]*University of Exeter, Living Systems Institute and College of Engineering Mathematics and Physical Sciences, Exeter, EX4 4QD, UK*

[*]Correspondence: fh111@cam.ac.uk, f.gielen@exeter.ac.uk.

Table of Contents

1. Data analysis to extract Michaelis-Menten kinetics from line camera data S2
2. Determination of the linear range of the absorbance readout in droplets S4
3. Supporting Figures S5
4. Supporting Tables S15
5. Supporting References S18

1. Data analysis to extract Michaelis-Menten kinetics from line camera data

The raw data read from the line camera as droplets pass through each detection point was converted into Michaelis-Menten plots using the following steps (see the github page https://github.com/fhlab/Line_detector_kinetics for code):

I. The time points corresponding to the end point of each gradient (i.e. time tag for the final droplet in the gradient) were identified visually by a gap that corresponds to the stopping of the flow after substrate injection. We subtracted the duration for substrate injection from these time points in order to calculate the actual start times of the gradients. Furthermore, we entered the number of droplets composing the gradient and overall gradient duration per detection point manually. Although these steps could be automated, it was found to be helpful in the following cases:

   - Imperfect monodispersity at the end of gradients (after transfer of the droplet maker to an oil well) leading to some larger droplets to not be counted.
   - Two droplets getting too close within the tubing and identified as a single drop, altering the overall droplet number.
   - Small differences seen between actual gradient duration (30 seconds) and duration read during the measurements due to local flow rate fluctuations, typically ±1 second.

II. Based on the input gradient times, droplet boundaries were identified from variations in the standard deviation of the moving average of the signal. By using the moving average, sudden signal spikes at the water-oil interfaces (i.e. spikes going above and below the signal corresponding to the actual droplet) are filtered out. This prevents incorrect droplet identification when the signal of the gradient is not monotonic due to the water-oil transitions.

III. After subtraction of the enzyme-only signal baseline, only points belonging to the gradient droplets were analyzed. Every gradient was fit using the following equation, as expected for second-order kinetics reactions:

$$y = ae^{b(t+c)} + d \quad (1)$$

Where *t* is time, *y* is the signal, and *a*, *b*, *c* and *d* are fitting constants.

IV. The software segmented the fit into the number of droplets counted in step I. This can be done as droplets are generated at fixed rate during substrate infusion. This step circumvented issues arising when droplets accidentally got too close, leading to

mismatches in tracking every individual reactor. Time tags were assigned by evaluating the mean time of every segment.

V. All the points corresponding to the same n^{th} droplets were connected and slopes extracted (i.e. reaction rates; reversing the droplet order every other gradient due to the reversal of flow direction).

VI. Enzyme concentration and substrate concentrations were inferred from the infusion/extraction flow rates and initial concentrations as described in a previous study[1] and the Michaelis-Menten parameters k_{cat} and K_M were obtained.

2. Determination of the linear range of the absorbance readout in droplets

We performed a linear data regression to determine the linear region of the calibration gradients, considering that the error in concentration can be assumed as a maximum time shift of 1 s between the estimated and actual start of the gradients (Figure S1). The resulting calibration curve indicated that linearity was observed up to just above 1 mM *p*NP.

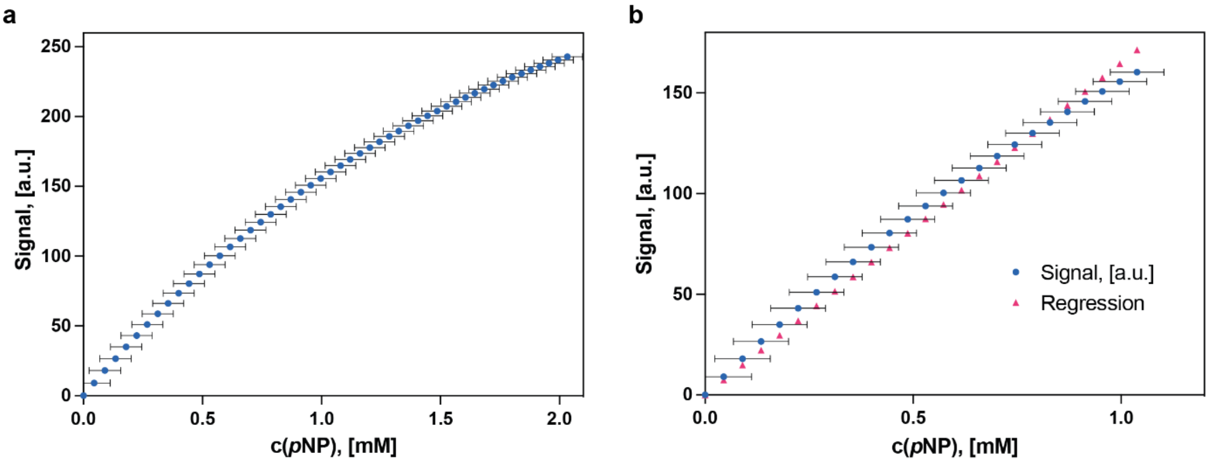

Figure S1: Calibration to determine the linear range of the detector. (**a)** Using a model *p*NP gradient, we obtained readings ranging from 0 to 2 mM *p*NP. **(b)** A linear regression confirms good linearity ($R^2 > 0.99$) up to 1 mM *p*NP. The error bars indicate the error in concentration resulting from a 1 s error range in time assignment. The example is shown for detection point 1 but is consistent across all 12 detection points.

After extracting the slope for the linear region, we obtained the limit of detection (LOD) for each detection point by calculating the standard deviation of the filtered signal level for the enzyme-only baseline. Using a confidence level of 95% (2 sigma above the determined background signal), we deduced that the LOD for every detection point ranged from ~5 to 18 µM (Figure S2).

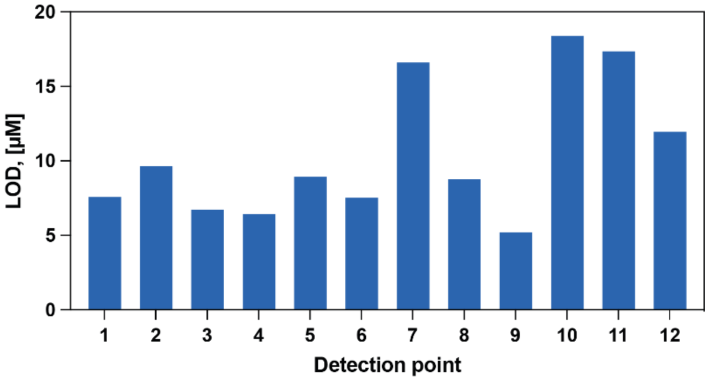

Figure S2. **Typical limits of detection for *p*-nitrophenol at the 12 detection points.** We ascribe the variations in LOD to imperfect incoming light power uniformity, and the imprecise alignment of tubing with respect to the threading holes, through which light propagates.

3. Supporting Figures

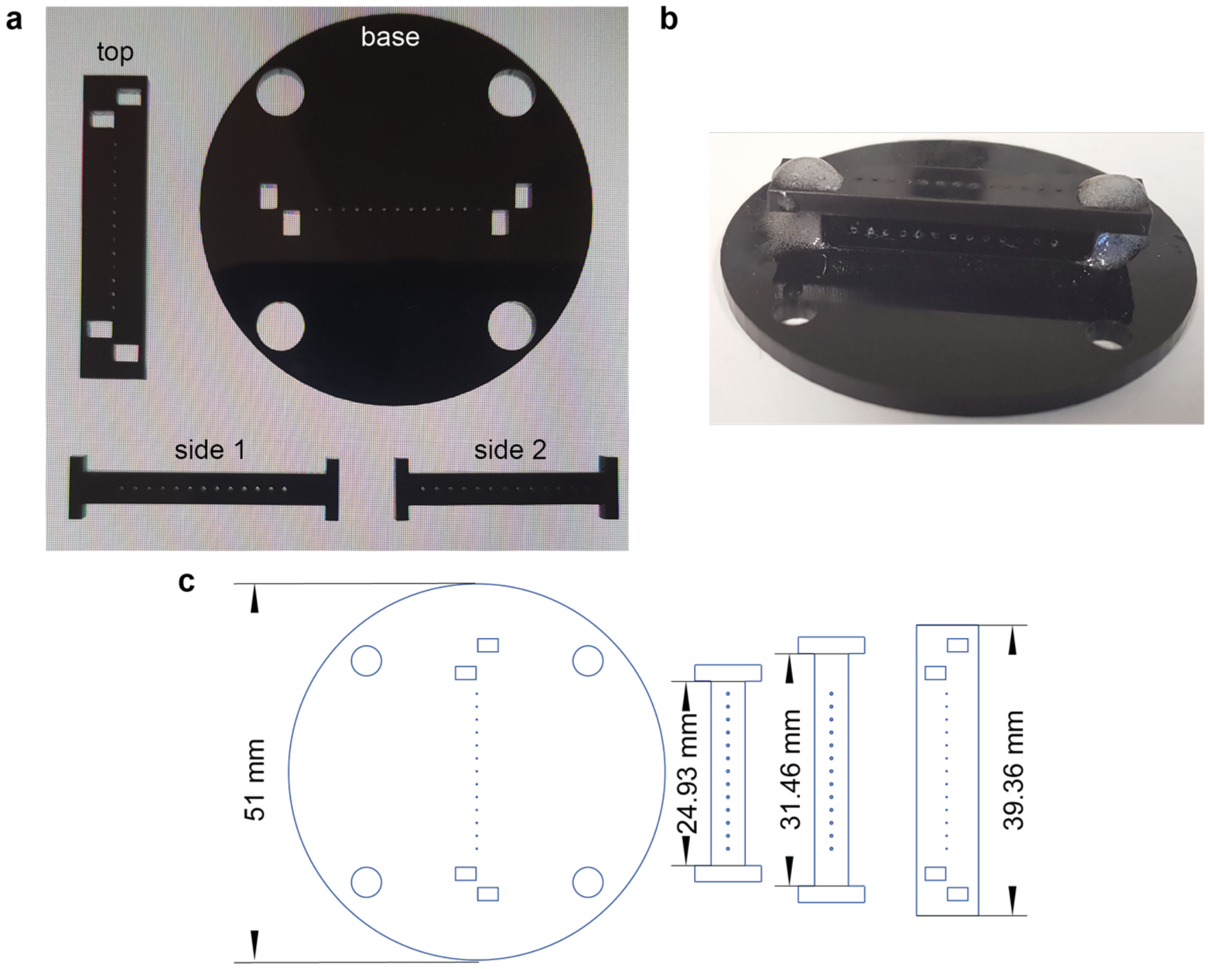

Figure S3. Fluidic connector aligning tubing for detection by the line camera. (a) Individual components made of black acrylic. The base, top and two side building blocks have evenly spaced holes (13 of which only 12 are used) that align with each other when assembled. **(b)** Assembled fluidic connector. The adaptor is placed on the line camera (see Figure 2, main article) and light from the collimated light sheet passes vertically through the holes from the top. Tubing is inserted horizontally through the holes in the two vertical side walls and pass through the light path. **(c)** Detailed drawing of the individual parts for the fluidic connector assembly. The holes have a diameter of 0.4 mm and the distance between the center of two holes is 1.75 mm. The corresponding design is provided as a pdf supporting file (and also available on https://openwetware.org/wiki/DropBase).

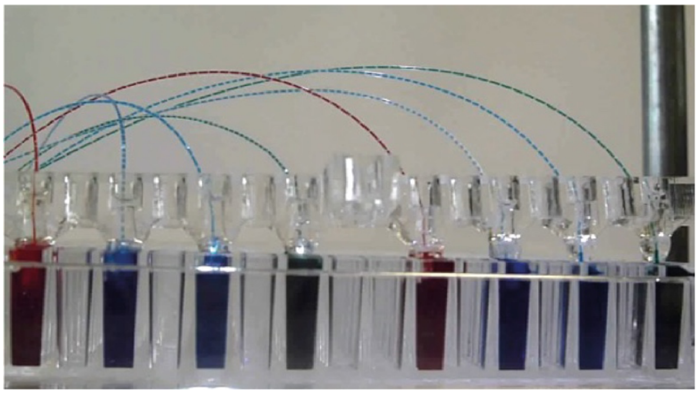

Figure S4. Towards automated sampling from microtiter plates. In this example, eight droplet generators operate in parallel, such that eight enzymatic reactions could be probed at the same time. The fluidic adaptor was made up of a main slab in clear acrylic with eight holes ~ 5 mm diameter, aligned with every other well of a 384-well plate. In each hole, the cut end of a gel loading tip was glued in its center. After insertion of the ultra-microbore PTFE tubing in the loading tip, oil can be pipetted into the sealed holes. A multi-rack syringe pump operates in withdrawal mode at constant flow rate across the eight fluidic lines.

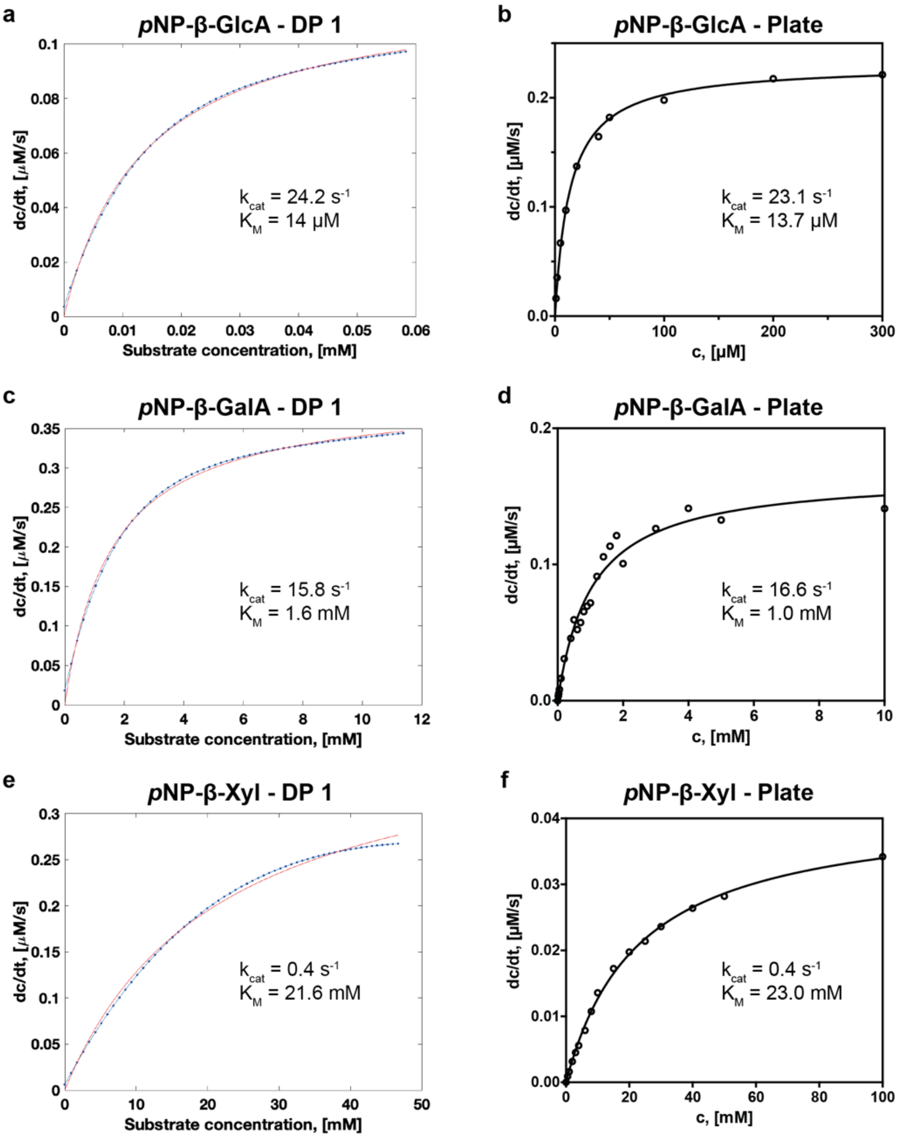

Figure S5: Side-by-side comparison of Michaelis-Menten kinetics determined from in-droplet measurements (for selected detection points) and microtiter plate measurements – part 1. Kinetic data were obtained for SN243 with *p*NP-β-GlcA **(a,b)**, *p*NP-β-GalA **(c,d)** and *p*NP-β-Xyl **(e,f)**. Resulting plots from absorbance detection at 405 nm in droplets **(a,c,e)** and in microtiter plates **(b,d,f)** were compared. DP: detection point.

Note: The differences in initial velocities (given as dc/dt in [μM/s]) between the two assay formats result from the usage of different enzyme concentrations. The microtiter plate data were obtained from Neun et al. (2022).[2]

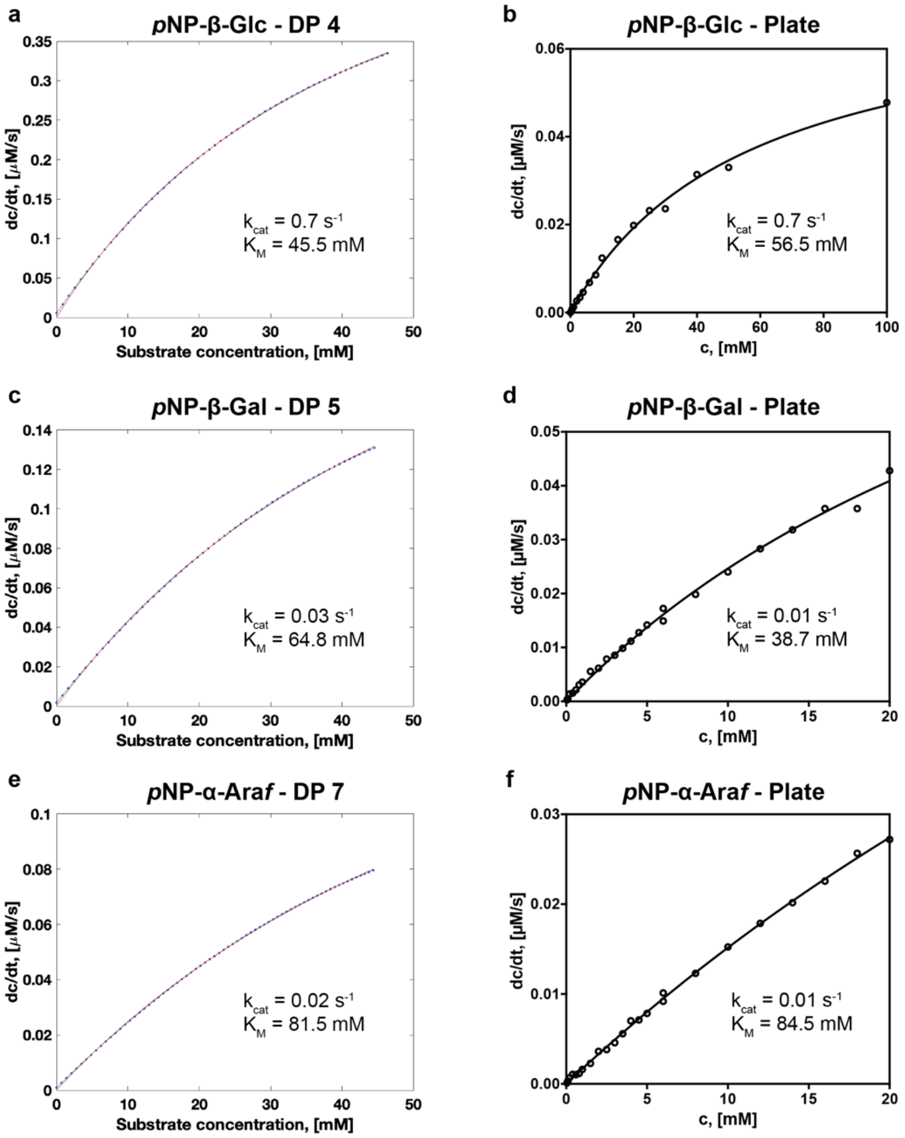

Figure S6: Side-by-side comparison of Michaelis-Menten kinetics determined from droplet (for selected detection points) and microtiter plate measurements– part 2. Kinetic data were obtained for SN243 with *p*NP-β-Glc **(a,b)**, *p*NP-β-Gal **(c,d)** and *p*NP-α-Ara*f* **(e,f)**. Resulting plots from absorbance detection at 405 nm in droplets **(a,c,e)** and in microtiter plates **(b,d,f)** were compared. Due to limited solubility of the substrates in aqueous solution the Michaelis-Menten datasets did not reach saturation and are extrapolations to a non-linear curve fit, implying that they carry a larger error than indicated based on the curve fit. The values for K_M and k_{cat} are therefore estimates derived from the initial phase of the Michaelis-Menten curve only.
DP: detection point.
Note: The differences in initial velocities (given as dc/dt in [μM/s]) between the two assay formats result from the usage of different enzyme concentrations. The microtiter plate data were obtained from Neun et al. (2022).[2]

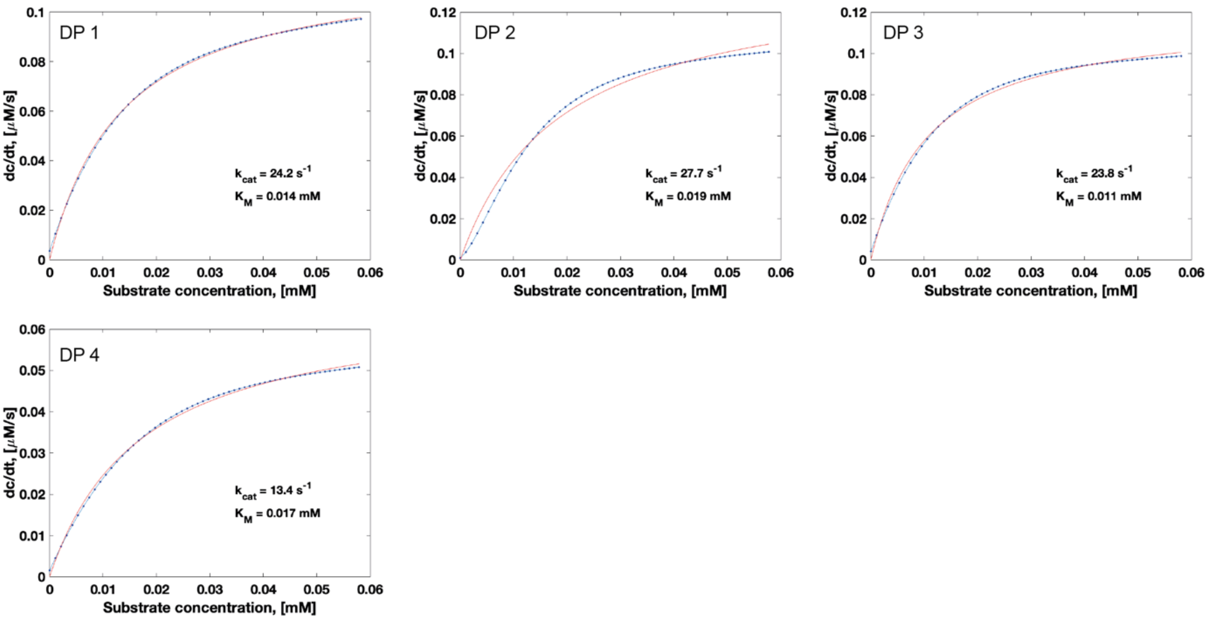

Figure S7: Individual plots for 4 detection points measured for the same droplet gradient for the determination of Michaelis-Menten kinetics of SN243 with *p*NP-β-GlcA. Initial velocities for the reaction extrapolated from droplet gradient measurements (blue) and fitting functions (red) are plotted for all detection points used to determine the average parameters indicated in Table 1 of the main manuscript. DP: detection point.

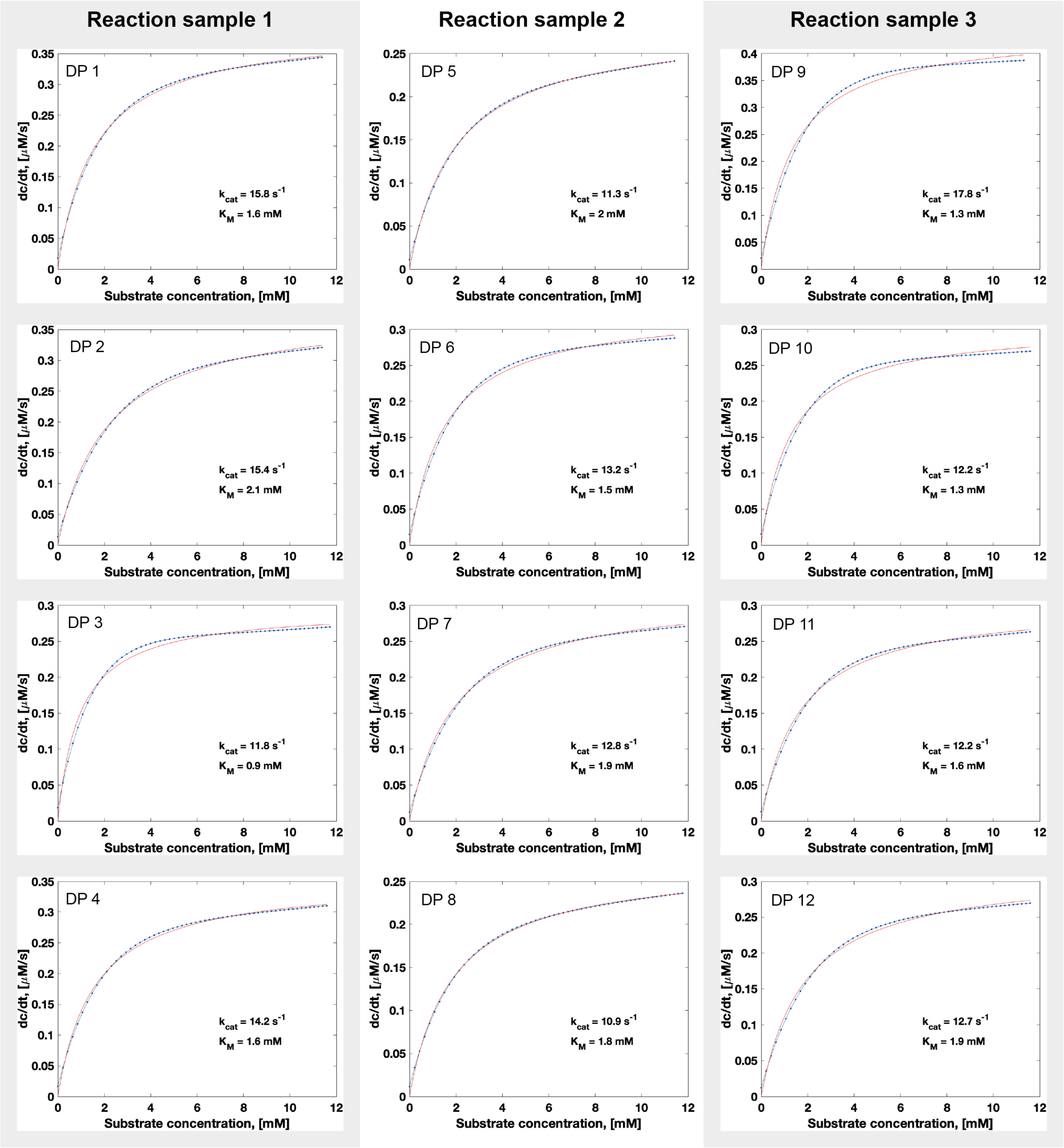

Figure S8: Individual plots for the 12 detection points for the accurate determination of Michaelis-Menten kinetics of SN243 with *p*NP-β-GalA in a single experiment. Detection points 1-4, 5-8 and 9-12 correspond to three distinct substrate concentration gradients. Initial velocities for the reaction extrapolated from droplet gradient measurements (blue) and fitting functions (red) are plotted for all detection points used to determine the average parameters indicated in Table 1 of the main manuscript. For sample 1 (detection points 1-4) K_M was determined with a mean value of 1.6 ± 0.5 mM and k_{cat} with 14.3 ± 1.8 s^{-1}, for sample 2 (detection points 5-8) K_M was 1.8 ± 0.2 mM and k_{cat} 12.1 ± 1.1 s^{-1}, and for sample 3 K_M was 1.5 ± 0.3 mM and k_{cat} 13.7 ± 2.7 s^{-1} (indicated errors are standard deviations. This results in an overall relative standard deviation of 21% in K_M and 15% in k_{cat} across three replicates. DP: detection point.

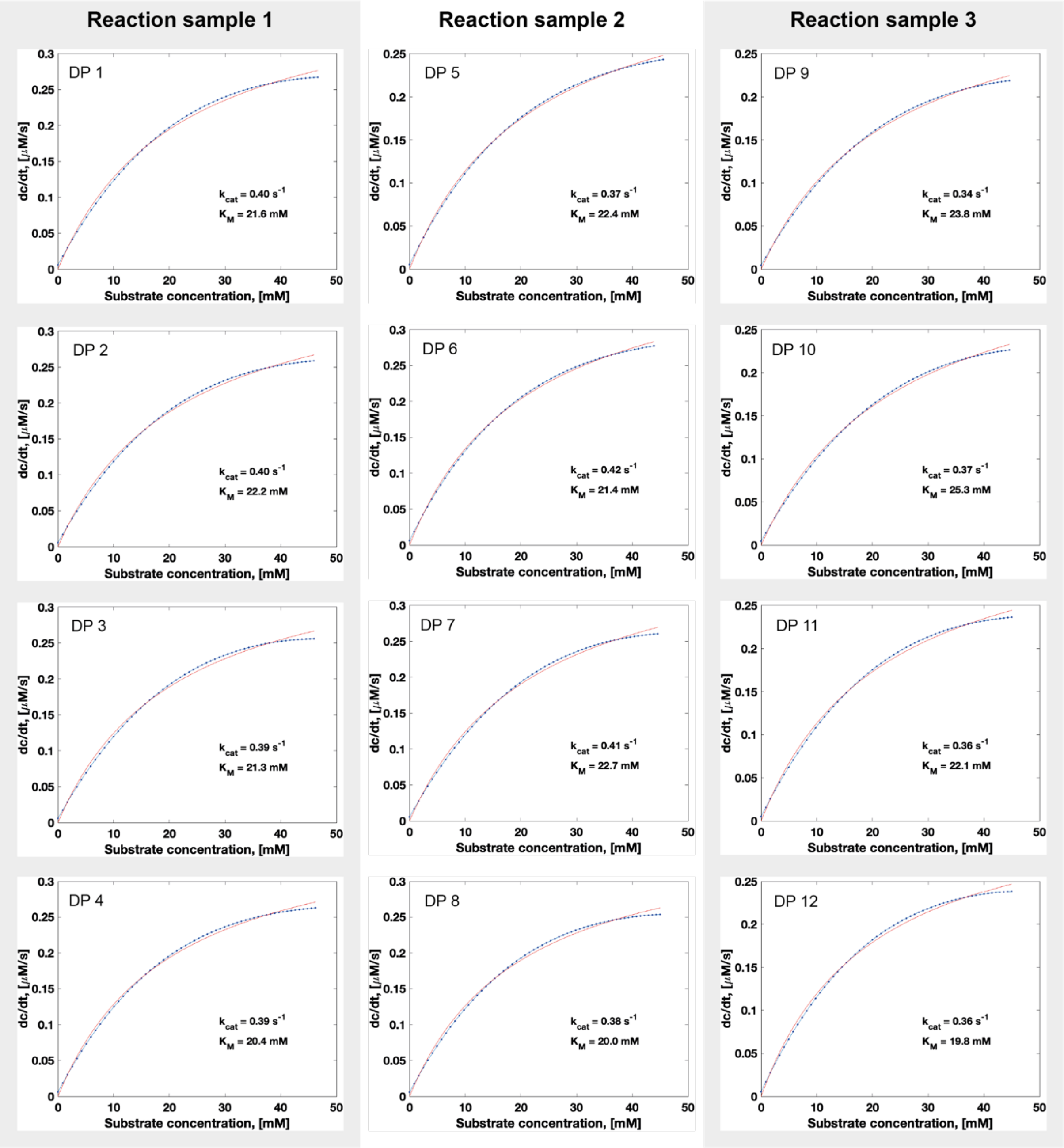

Figure S9: Individual plots for 12 detection points for the accurate determination of Michaelis-Menten kinetics of SN243 with *p*NP-β-Xyl in a single experiment. Detection points 1-4, 5-8 and 9-12 correspond to three distinct substrate concentration gradients. Initial velocities for the reaction extrapolated from droplet gradient measurements (blue) and fitting functions (red) are plotted for all detection points used to determine the average parameters indicated in Table 1 of the main manuscript. For sample 1 (detection points 1-4) K_M was determined with a mean value of 21.4 ± 0.8 mM and k_{cat} with 0.4 ± 0.007 s^{-1}, for sample 2 (detection points 5-8) K_M was 21.6 ± 1.2 mM and k_{cat} 0.4 ± 0.02 s^{-1}, and for sample 3 K_M was 22.7 ± 2.4 mM and k_{cat} 0.4 ± 0.01 s^{-1} (indicated errors are standard deviations. This results in an overall relative standard deviation of 7% in K_M and 4% in k_{cat} across three replicates.
DP: detection point.

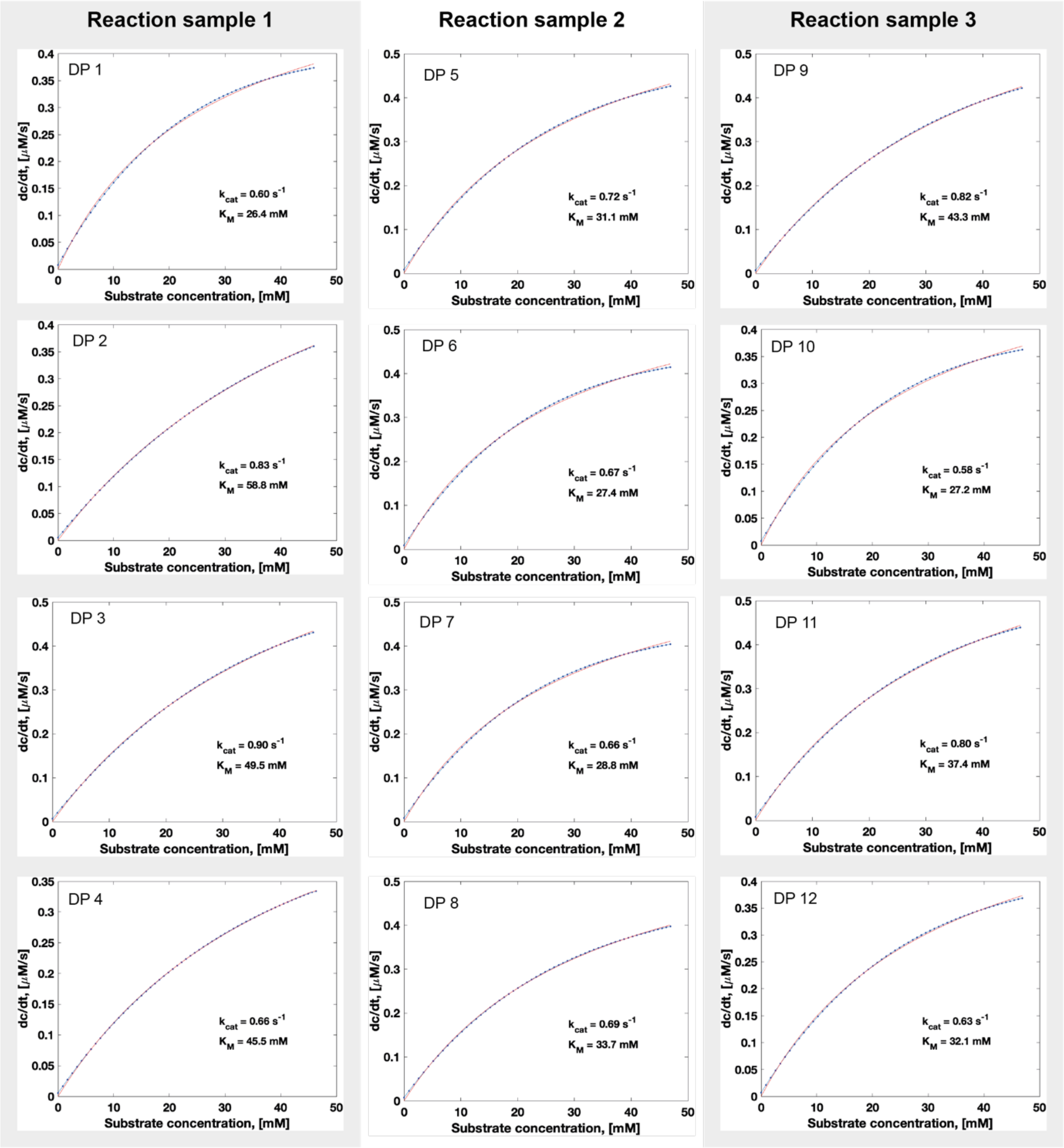

Figure S10: Individual plots for 12 detection points for the determination of Michaelis-Menten kinetics of SN243 with *p*NP-β-Glc in a single experiment. Detection points 1-4, 5-8 and 9-12 correspond to three distinct substrate concentration gradients. Initial velocities for the reaction extrapolated from droplet gradient measurements (blue) and fitting functions (red) are plotted for all detection points used to determine the average parameters indicated in Table 1 of the main manuscript. For sample 1 (detection points 1-4) K_M was determined with a mean value of 45.0 ± 13.6 mM and k_{cat} with 0.7 ± 0.1 s^{-1}, for sample 2 (detection points 5-8) K_M was 30.2 ± 2.7 mM and k_{cat} 0.7 ± 0.02 s^{-1}, and for sample 3 K_M was 35.0 ± 6.9 mM and k_{cat} 0.7 ± 0.1 s^{-1} (indicated errors are standard deviations. This results in an overall relative standard deviation of 24% in K_M and 15% in k_{cat} across three replicates. Note that due to limited solubility of the substrate in aqueous solution the shown Michaelis-Menten kinetics are extrapolations, and the indicated values (K_M and k_{cat}) are only estimates derived from the initial phase of the Michaelis-Menten curve.
DP: detection point.

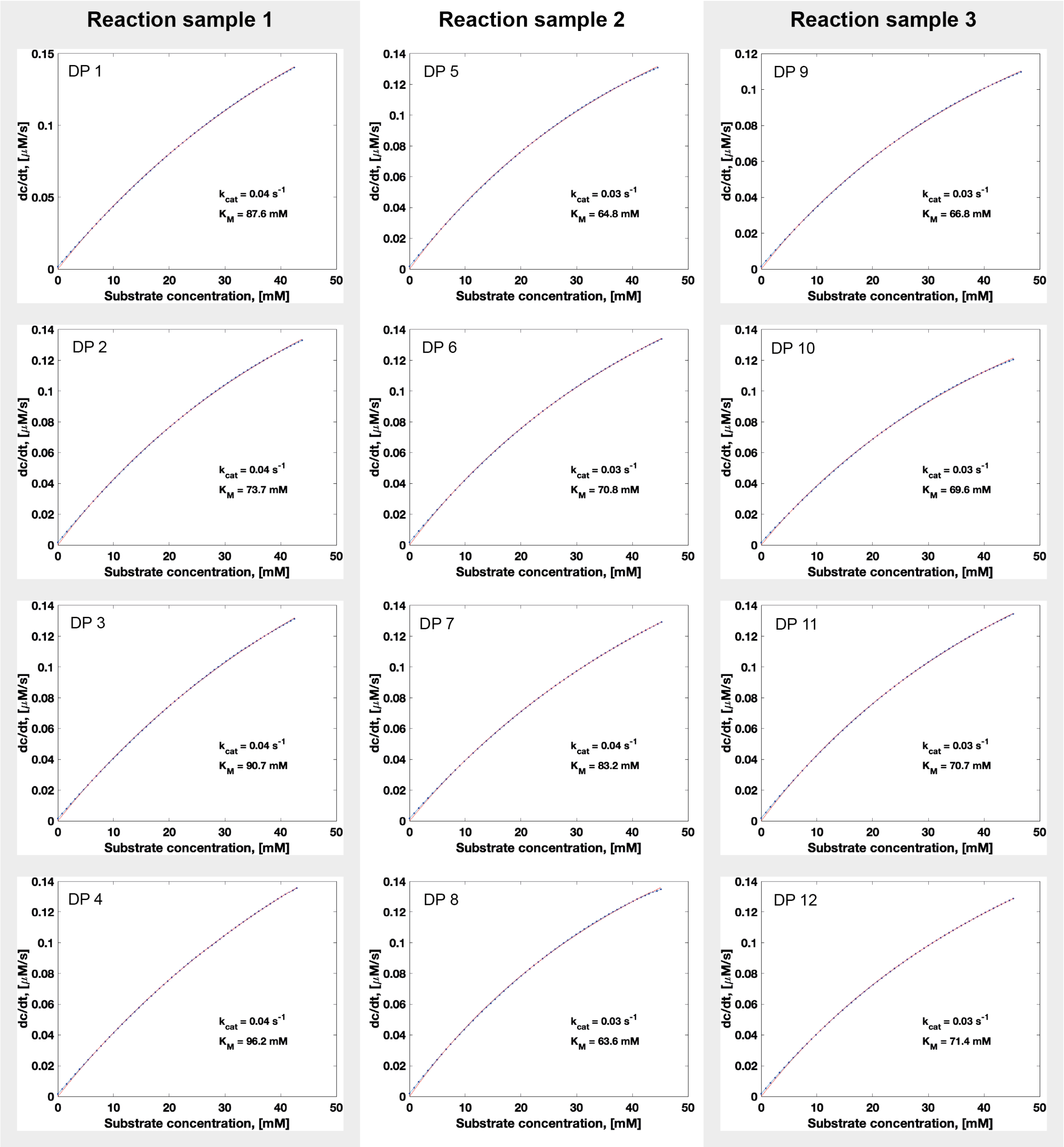

Figure S11: Individual plots for 12 detection points for the determination of Michaelis-Menten kinetics of SN243 with *p*NP-β-Gal in a single experiment. Detection points 1-4, 5-8 and 9-12 correspond to three distinct substrate concentration gradients. Initial velocities for the reaction extrapolated from droplet gradient measurements (blue) and fitting functions (red) are plotted for all detection points used to determine the average parameters indicated in Table 1 of the main manuscript. Fitting the incomplete non-linear Michaelis-Menten curves based on a dataset that has not reached saturation, implies that the errors are larger than those indicated based on averaging and fitting. For sample 1 (detection points 1-4) K_M was determined with a mean value of 87.0 ± 9.6 mM and k_{cat} with 4.2 x 10^{-2} ± 0.4 x 10^{-2} s^{-1}, for sample 2 (detection points 5-8) K_M was 70.6 ± 9.0 mM and k_{cat} 3.4 x 10^{-2} ± 0.2 x 10^{-2} s^{-1}, and for sample 3 K_M was 69.6 ± 2.0 mM and k_{cat} 3.1 x 10^{-2} ± 0.3 x 10^{-2} s^{-1} (indicated errors are standard deviations. This results in an overall relative standard deviation of 10% in K_M and 9% in k_{cat} across three replicates. Note that due to limited solubility of the substrate in aqueous solution the shown Michaelis-Menten kinetics are extrapolations, and the indicated values (K_M and k_{cat}) are only estimates derived from the initial phase of the Michaelis-Menten curve.
DP: detection point.

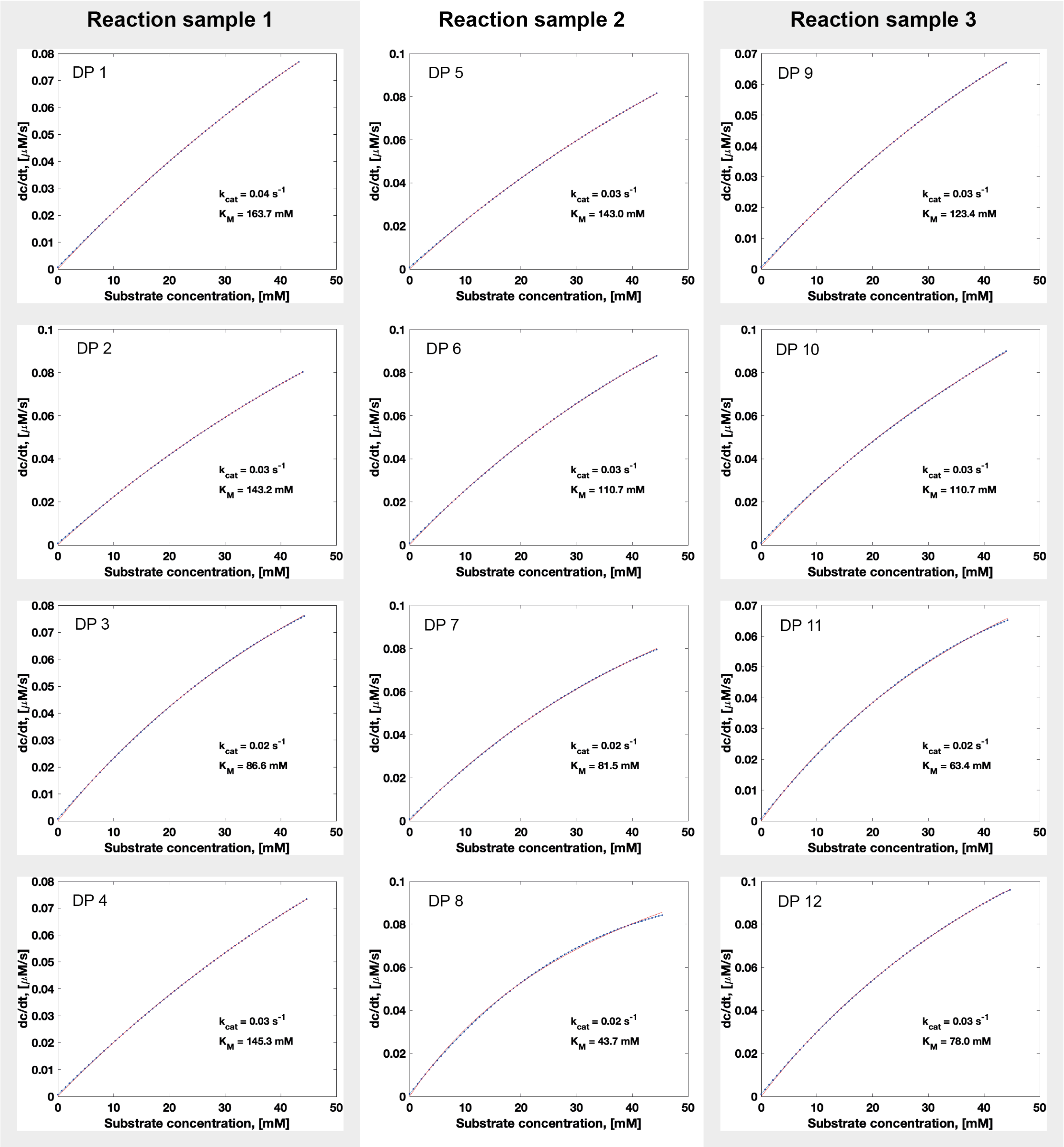

Figure S12: Individual plots for 12 detection points for the determination of Michaelis-Menten kinetics of SN243 with *p*NP-α-Ara*f* in a single experiment. Detection points 1-4, 5-8 and 9-12 correspond to three distinct substrate concentration gradients. Initial velocities for the reaction extrapolated from droplet gradient measurements (blue) and fitting functions (red) are plotted for all detection points used to determine the average parameters indicated in Table 1 of the main manuscript. Fitting the incomplete non-linear Michaelis-Menten curves based on a dataset that has not reached saturation, implies that the errors are larger than those indicated based on averaging and fitting. For sample 1 (detection points 1-4) K_M was determined with a mean value of 134.7 ± 33.3 mM and k_{cat} with 3.1 x 10^{-2} ± 0.6 x 10^{-2} s^{-1}; for sample 2 (detection points 5-8) K_M was 94.7 ± 42.3 mM and k_{cat} 2.6 x 10^{-2} ± 0.8 x 10^{-2} s^{-1}, and for sample 3 K_M was 93.9 ± 27.9 mM and k_{cat} 2.5 x 10^{-2} ± 0.6 x 10^{-2} s^{-1} (the errors indicated are standard deviations). This results in an overall relative standard deviation of 32% in K_M and 25% in k_{cat} across three replicates. Note that due to limited solubility of the substrate in aqueous solution the Michaelis-Menten kinetics shown are extrapolations, and the values for K_M and k_{cat} are estimates only, derived from the initial phase of the Michaelis-Menten curve. DP: detection point.

4. Supporting Tables

Table S1: Microfluidic systems for kinetic analysis using droplets generated and measured in continuous flow devices.

Ref.	Concentrations one-by-one or gradient?	Readout	Different reactions in parallel	Individual droplets or averaging over many droplets?	Time points from same or separate experiment	Datapoints per kinetic dataset	Datapoints per v_0 determination	Duration of operation	Reaction time scale	Kinetic analysis	Difficulty
[3]	One-by-one	Fluorescence intensity	1	Averaging	Same	4	~10	min-h	Fast	Pre-steady state analysis	++
[4]						10-15					
[5, 6]		Electrochemistry			Separate	5					+++
[7]	Gradient (4 concentrations by laminar flow)	Fluorescence intensity			Same	10			Slow	Steady-state analysis	+++
[8]	One-by-one	Absorbance (LEDs)				6	7				++
[9]		Photothermal interferometry				5	4				
[10]	gradient (by flow rate variation)	Fluorescence intensity		Individually		~10^3	35		Fast		
[11]	Inhibitor gradient (generated in capillary prior to droplet formation)	Electrochemistry			n.a.	40-50	1		Slow	Inhibitor potency measurement	+++
[12]	One-by-one	Absorbance optical fiber-based)		Averaging		7					++
[13]	Inhibitor gradient (generated in capillary prior to droplet formation)	Laser-induced fluorescence				28					++

*(low concentrations/linear range of Michaelis-Menten plot not captured); n.a.: not applicable.

Table S2: Microfluidic systems for kinetic analysis using droplets in segmented flow.

Ref.	Concentrations one-by-one or gradient?	Readout	Different reactions in parallel	Individual droplets or averaging over many droplets?	Time points from same or separate experiment	Datapoints per kinetic dataset	Datapoints per v_0 determination	Duration of operation	Reaction time scale	Kinetic analysis	Difficulty
[14]	Inhibitor gradient in droplets-on-demand (pre-pipetted microtiter plate)	Laser-induced fluorescence	1	Individually	Same	7	7	hours	Slow	Inhibitor potency measurement	+++
[15]	Inhibitor gradient (5 concentrations by flow ratio adjustment)			Averaging		5 substrate and 5 inhibitor concentrations	6	min-h			
[16]	gradient in droplets-on-demand (by coalescing defined numbers of reagent droplets)	Fluorescence intensity		Individually		32	12				
[17]	concentration gradient in droplets-on-demand (generated in capillary prior to droplet formation)		102			24	6	hours		Compound screening	+++
[18]	gradient in droplets-on-demand (by valve-based system)		2			~5	10	min-h		Steady-state analysis	+++
[19]	gradient in droplets-on-demand (6 concentrations from separate syringes)		1				20		Fast & slow	(Pre-)steady-state analysis	
[1]	concentration gradient in droplets-on-demand (by merging droplets of different concentration and volume ratios)	Absorbance				24	10		Slow	Steady-state analysis	+
[20]	Concentration gradient in droplets-on-demand (droplets generated while changing concentration in source well)					150	6				
This study			12			60					

Table S3: Droplet-free microfluidic systems for kinetic analysis.

<table>
<tr><th>Ref.</th><th>Concentrations one-by-one or gradient?</th><th>Readout</th><th>Different reactions in parallel</th><th>Individual measurements or averaging over time?</th><th>Time points from same or separate experiment</th><th>Datapoints per kinetic dataset</th><th>Datapoints per v_0 determination</th><th>Duration of operation</th><th>Reaction time scale</th><th>Kinetic analysis</th><th>Difficulty</th></tr>
<tr><td>[21]</td><td rowspan="3">One-by-one</td><td>Laser-induced fluorescence</td><td rowspan="6">1</td><td rowspan="3">Averaging</td><td>Same</td><td rowspan="3"><10</td><td>15-20</td><td rowspan="2">min-h</td><td rowspan="7">slow</td><td>Steady-state analysis</td><td>++</td></tr>
<tr><td>[22]</td><td>Fluorescence intensity</td><td rowspan="2">Separate</td><td rowspan="2">~5</td><td rowspan="2">Reuse of enzyme for steady-state analysis</td><td rowspan="2">++</td></tr>
<tr><td>[23]</td><td>Electrochemistry</td><td rowspan="2">hours</td></tr>
<tr><td>[24]</td><td>Concentration gradient (in microprocessors)</td><td rowspan="2">Fluorescence intensity</td><td rowspan="2">Individually</td><td rowspan="4">Same</td><td>11</td><td>~30</td><td rowspan="3">Steady-state analysis</td><td>++++</td></tr>
<tr><td>[25]</td><td rowspan="3">One-by-one</td><td>12</td><td>60</td><td>min-h</td><td>+</td></tr>
<tr><td>[26]</td><td>Infrared</td><td>Averaging</td><td>9</td><td>8</td><td>hours</td><td>+++</td></tr>
<tr><td>[27]</td><td>Fluorescence intensity</td><td>1500</td><td>Individually</td><td>10-15</td><td>15-20</td><td>days</td><td>Expression, purification and steady-state analysis on one chip</td><td>++++</td></tr>
</table>

5. Supporting References

(1) Gielen, F.; van Vliet, L.; Koprowski, B. T.; Devenish, S. R. A.; Fischlechner, M.; Edel, J. B.; Niu, X.; deMello, A. J.; Hollfelder, F. A Fully Unsupervised Compartment-on-Demand Platform for Precise Nanoliter Assays of Time-Dependent Steady-State Enzyme Kinetics and Inhibition. *Anal. Chem.* **2013**, *85* (9), 4761-4769. DOI: 10.1021/ac400480z.

(2) Neun, S.; Brear, P.; Campbell, E.; Tryfona, T.; El Omari, K.; Wagner, A.; Dupree, P.; Hyvönen, M.; Hollfelder, F. Functional Metagenomic Screening Identifies an Unexpected β-Glucuronidase. *Nat. Chem. Biol.* **2022**. DOI: 10.1038/s41589-022-01071-x.

(3) Song, H.; Ismagilov, R. F. Millisecond Kinetics on a Microfluidic Chip Using Nanoliters of Reagents. *J. Am. Chem. Soc.* **2003**, *125* (47), 14613-14619. DOI: 10.1021/ja0354566.

(4) Hess, D.; Dockalova, V.; Kokkonen, P.; Bednar, D.; Damborsky, J.; deMello, A.; Prokop, Z.; Stavrakis, S. Exploring Mechanism of Enzyme Catalysis by On-Chip Transient Kinetics Coupled with Global Data Analysis and Molecular Modeling. *Chem* **2021**, *7* (4), 1066-1079. DOI: 10.1016/j.chempr.2021.02.011.

(5) Han, Z.; Li, W.; Huang, Y.; Zheng, B. Measuring Rapid Enzymatic Kinetics by Electrochemical Method in Droplet-Based Microfluidic Devices with Pneumatic Valves. *Anal. Chem.* **2009**, *81* (14), 5840-5845. DOI: 10.1021/ac900811y.

(6) Han, Z.; Chang, Y. Y.; Au, S. W. N.; Zheng, B. Measuring Rapid Kinetics by a Potentiometric Method in Droplet-Based Microfluidic Devices. *Chem. Commun.* **2012**, *48* (10), 1601-1603. DOI: 10.1039/C1CC12383A.

(7) Damean, N.; Olguin, L. F.; Hollfelder, F.; Abell, C.; Huck, W. T. S. Simultaneous Measurement of Reactions in Microdroplets Filled by Concentration Gradients. *Lab Chip* **2009**, *9* (12), 1707-1713. DOI: 10.1039/B821021G.

(8) Hassan, S.-u.; Nightingale, A. M.; Niu, X. Continuous Measurement of Enzymatic Kinetics in Droplet Flow for Point-of-Care Monitoring. *Analyst* **2016**, *141* (11), 3266-3273. DOI: 10.1039/C6AN00620E.

(9) Maceiczyk, R. M.; Hess, D.; Chiu, F. W. Y.; Stavrakis, S.; deMello, A. J. Differential Detection Photothermal Spectroscopy: Towards Ultra-Fast and Sensitive Label-Free Detection in Picoliter & Femtoliter Droplets. *Lab Chip* **2017**, *17* (21), 3654-3663. DOI: 10.1039/C7LC00946A.

(10) Hess, D.; Rane, A.; deMello, A. J.; Stavrakis, S. High-Throughput, Quantitative Enzyme Kinetic Analysis in Microdroplets Using Stroboscopic Epifluorescence Imaging. *Anal. Chem.* **2015**, *87* (9), 4965-4972. DOI: 10.1021/acs.analchem.5b00766.

(11) Gu, S.; Lu, Y.; Ding, Y.; Li, L.; Zhang, F.; Wu, Q. Droplet-Based Microfluidics for Dose–Response Assay of Enzyme Inhibitors by Electrochemical Method. *Anal. Chim. Acta* **2013**, *796*, 68-74. DOI: 10.1016/j.aca.2013.08.016.

(12) Mao, Z.; Guo, F.; Xie, Y.; Zhao, Y.; Lapsley, M. I.; Wang, L.; Mai, J. D.; Costanzo, F.; Huang, T. J. Label-Free Measurements of Reaction Kinetics Using a Droplet-Based Optofluidic Device. *SLAS Technol.* **2015**, *20* (1), 17-24. DOI: 10.1177/2211068214549625.

(13) Miller, O. J.; Harrak, A. E.; Mangeat, T.; Baret, J.-C.; Frenz, L.; Debs, B. E.; Mayot, E.; Samuels, M. L.; Rooney, E. K.; Dieu, P.; et al. High-Resolution Dose-Response Screening using Droplet-Based Microfluidics. *Proc. Natl. Acad. Sci.* **2012**, *109* (2), 378-383. DOI: 10.1073/pnas.1113324109.

(14) Clausell-Tormos, J.; Griffiths, A. D.; Merten, C. A. An Automated Two-Phase Microfluidic System for Kinetic Analyses and the Screening of Compound Libraries. *Lab Chip* **2010**, *10* (10), 1302-1307. DOI: 10.1039/B921754A.

(15) Sjostrom, S. L.; Joensson, H. N.; Svahn, H. A. Multiplex Analysis of Enzyme Kinetics and Inhibition by Droplet Microfluidics Using Picoinjectors. *Lab Chip* **2013**, *13* (9), 1754-1761. DOI: 10.1039/C3LC41398E.

(16) Lee, B.; Jin, S. H.; Noh, Y.-M.; Jeong, S.-G.; Jeong, H.-H.; Lee, C.-S. Scalable Static Droplet Array for Biochemical Assays Based on Concentration Gradients. *Sens. Actuators, B* **2018**, *273*, 1572-1578. DOI: 10.1016/j.snb.2018.07.076.

(17) Wei, Y.; Zhu, Y.; Fang, Q. Nanoliter Quantitative High-Throughput Screening with Large-Scale Tunable Gradients Based on a Microfluidic Droplet Robot under Unilateral Dispersion Mode. *Anal. Chem.* **2019**, *91* (8), 4995-5003. DOI: 10.1021/acs.analchem.8b04564.

(18) Jambovane, S.; Kim, D. J.; Duin, E. C.; Kim, S.-K.; Hong, J. W. Creation of Stepwise Concentration Gradient in Picoliter Droplets for Parallel Reactions of Matrix Metalloproteinase II and IX. *Anal. Chem.* **2011**, *83* (9), 3358-3364. DOI: 10.1021/ac103217p.

(19) Fradet, E.; Bayer, C.; Hollfelder, F.; Baroud, C. N. Measuring Fast and Slow Enzyme Kinetics in Stationary Droplets. *Anal. Chem.* **2015**, *87* (23), 11915-11922. DOI: 10.1021/acs.analchem.5b03567.

(20) Gielen, F.; Buryska, T.; Vliet, L. V.; Butz, M.; Damborsky, J.; Prokop, Z.; Hollfelder, F. Interfacing Microwells with Nanoliter Compartments: A Sampler Generating High-Resolution Concentration Gradients for Quantitative Biochemical Analyses in Droplets. *Anal. Chem.* **2015**, *87* (1), 624-632. DOI: 10.1021/ac503336g.

(21) Hadd, A. G.; Raymond, D. E.; Halliwell, J. W.; Jacobson, S. C.; Ramsey, J. M. Microchip Device for Performing Enzyme Assays. *Anal. Chem.* **1997**, *69* (17), 3407-3412. DOI: 10.1021/ac970192p.

(22) Seong, G. H.; Heo, J.; Crooks, R. M. Measurement of Enzyme Kinetics Using a Continuous-Flow Microfluidic System. *Anal. Chem.* **2003**, *75* (13), 3161-3167. DOI: 10.1021/ac034155b.

(23) Wang, C.; Li, S.-J.; Wu, Z.-Q.; Xu, J.-J.; Chen, H.-Y.; Xia, X.-H. Study on the Kinetics of Homogeneous Enzyme Reactions in a Micro/Nanofluidics Device. *Lab Chip* **2010**, *10* (5), 639-646. DOI: 10.1039/B915762J.

(24) Jambovane, S.; Duin, E. C.; Kim, S.-K.; Hong, J. W. Determination of Kinetic Parameters, Km and kcat, with a Single Experiment on a Chip. *Anal. Chem.* **2009**, *81* (9), 3239-3245. DOI: 10.1021/ac8020938.

(25) Rembeza, E.; Engqvist, M. K. M. Adaptation of a Microfluidic qPCR System for Enzyme Kinetic Studies. *ACS Omega* **2021**, *6* (3), 1985-1990. DOI: 10.1021/acsomega.0c04918.

(26) Srisa-Art, M.; Noblitt, S. D.; Krummel, A. T.; Henry, C. S. IR-Compatible PDMS Microfluidic Devices for Monitoring of Enzyme Kinetics. *Anal. Chim. Acta* **2018**, *1021*, 95-102. DOI: 10.1016/j.aca.2018.03.006.

(27) Markin, C. J.; Mokhtari, D. A.; Sunden, F.; Appel, M. J.; Akiva, E.; Longwell, S. A.; Sabatti, C.; Herschlag, D.; Fordyce, P. M. Revealing Enzyme Functional Architecture via High-Throughput Microfluidic Enzyme Kinetics. *Science* **2021**, *373* (6553), eabf8761. DOI: 10.1126/science.abf8761.
